# Frailty in the context of kidney transplantation

**DOI:** 10.1590/2175-8239-JBN-2024-0048en

**Published:** 2024-09-27

**Authors:** Tainá Veras de Sandes-Freitas, Raoni de Oliveira Domingues-da-Silva, Helady Sanders-Pinheiro

**Affiliations:** 1 Universidade Federal do Ceará, Faculdade de Medicina, Fortaleza, CE, Brazil.; 2 Hospital Geral de Fortaleza, Fortaleza, CE, Brazil; 3 Universidade Federal de Juiz de Fora, Faculdade de Medicina, Núcleo Interdisciplinar de Estudos e Pesquisas em Nefrologia (NIEPEN), Juiz de Fora, MG, Brazil.; 4 Universidade Federal de Juiz de Fora, Hospital Universitário, Serviço de Transplante Renal, Juiz de Fora, MG, Brazil.

**Keywords:** Frailty, Kidney Transplant, Chronic Kidney Disease, Incidence, Risk Factors, Access to Health Services

## Abstract

Frailty, defined as an inappropriate response to stressful situations due to the loss of physiological reserve, was initially described in the elderly population, but is currently being identified in younger populations with chronic diseases, such as chronic kidney disease. It is estimated that about 20% of patients are frail at the time of kidney transplantation (KT), and there is great interest in its potential predictive value for unfavorable outcomes. A significant body of evidence has been generated; however, several areas still remain to be further explored. The pathogenesis is poorly understood and limited to the extrapolation of findings from other populations. Most studies are observational, involving patients on the waiting list or post-KT, and there is a scarcity of data on long-term evolution and possible interventions. We reviewed studies, including those with Brazilian populations, assessing frailty in the pre- and post-KT phases, exploring pathophysiology, associated factors, diagnostic challenges, and associated outcomes, in an attempt to provide a basis for future interventions.

## Introduction

With advances in the efficiency of immunosuppress­ants, in the selection of recipients (HLA typing techniques and identification of donor-specific antibodies), and better management of infections, the long-term survival of kidney grafts has significantly improved. However, this improvement is not consistent for all patient groups^1^. Potential explanations include the changing epidemiological profile of kidney transplant (KT) candidates. Even though patients on KT lists are the healthiest, the mean age of patients has increased, as have the comorbidities^2,3^. A more accurate means of assessing clinical conditions at the time of KT is identifying the presence of frailty^4^. Initially developed to properly distinguish physiological from chronological age in older individuals, the diagnosis of frailty syndrome has been used to more accurately identify vulnerability to undesirable outcomes beyond mere age or the presence of morbidities. More recently, it has been identified and studied in non-elderly populations, such as patients with chronic kidney disease (CKD) and KT^4,5^.

Frailty is an entity characterized by an inadequate response to stressful situations due to the loss of physiological reserve^6^. Although it overlaps with the presence of morbidities and physical limitations, it is an independent risk factor for poorer outcomes in CKD and KT patients^7,8^. Identifying frailty and potential associated factors could promote early approaches, both for the development of preventive measures in the management of candidates and for post-KT period^4,9^.

We conducted a review of the SciELO, PubMed and LILACS databases using the following terms: “frailty” and “kidney transplantation”, in the period from 2012 to 2024. A secondary search was also carried out based on articles selected from the primary search that met the study’s objectives.

Definition, Pathogenesis, Risk Factors and Associated Conditions

Frailty is a multifactorial condition resulting from dysregulation or deterioration of homeostasis and physiological reserves, and increased vulnerability to both environmental and internal stressors. Frailty syndrome may be understood either as a purely physical condition (physical frailty phenotype) or as a multidimensional vulnerability syndrome (accumulation of deficits)^6^.

Among the fundamental components of the physical frailty phenotype is sarcopenia, which is characterized by a reduction in muscle mass, strength, and function, commonly associated with aging. However, sarcopenia alone does not explain frailty, and these conditions should not be understood as synonymous, despite often being associated. Factors other than sarcopenia contribute to the pathogenesis of physical frailty syndrome, such as inactivity, malnutrition, chronic inflammation, hormonal and immune system dysregulation, and other clinical conditions^10^. In addition to the physical component, an individual may be considered frail due to other vulnerabilities, such as cognitive decline, impairment of psychological (e.g. depression) and social (e.g. low education, low income, and lack of support network) components^11^.

The frailty syndrome was first described in the context of aging and the resulting impairment of systemic functions. In fact, this condition is more prevalent among the elderly; however, it may also be observed in non-elderly individuals presenting with certain clinical conditions, notably chronic degenerative diseases such as CKD. The precise mechanism by which CKD is associated with frailty, regardless of age, remains unclear. However, it is likely that chronic inflammation, anemia, malnutrition, sarcopenia, and inactivity are implicated in its pathogenesis^12^.

In addition to aging and chronic diseases, genetic susceptibility and socio-environmental conditions play a significant role in determining frailty. Among the genetic factors, we would highlight DNA repair and reduced telomere erosion rates, which are fundamental functions performed by the p53 and p16 protein complexes. Studies indicate that the p53 protein is associated with age, as evidenced by polymorphism investigations in both men and women^13^. Additionally, overexpression of the p16 protein in mice has been shown to exert an anti-aging effect, suggesting an association between DNA protection and repair genes, and frailty^13^. Conversely, Kumar et al.^14^ identified a significant association between low levels of sirtuins, a family of proteins with enzymatic deacetylation activity, and frailty.

Regarding socio-environmental conditions, limited access to health resources and inadequate social support could increase vulnerability to frailty, irrespective of age group. In a cross-sectional study comprising 727 women, it was observed that women with up to elementary school education had a three-fold greater likelihood of being frail compared to individuals with higher levels of education, regardless of age, race, or health insurance^15^.

Other conditions commonly associated with frailty syndrome include mental disorders such as depression, cognitive dysfunction, sleep disturbances, and loss of functional capacity to perform basic and instrumental activities of daily living^16,17,18,19^. Furthermore, polypharmacy (regular use of ≥ 5 medications) and hyperpolypharmacy (≥ 10 medications) have been described in this group of frail patients, including among KT recipients^20^.

Biological sex is also a relevant factor in assessing the frailty phenotype in KT candidates, with the prevalence of this condition being up to twice as high in females^21^. The mechanisms responsible for the greater risk of frailty in women remain to be further clarified. Interestingly, despite being more often diagnosed as frail, women with CKD in pre-dialysis treatment have lower mortality than men. This is known as the “sex paradox”, an effect not observed when considering patients on dialysis and after KT^22,23^.

**Figure 1.**
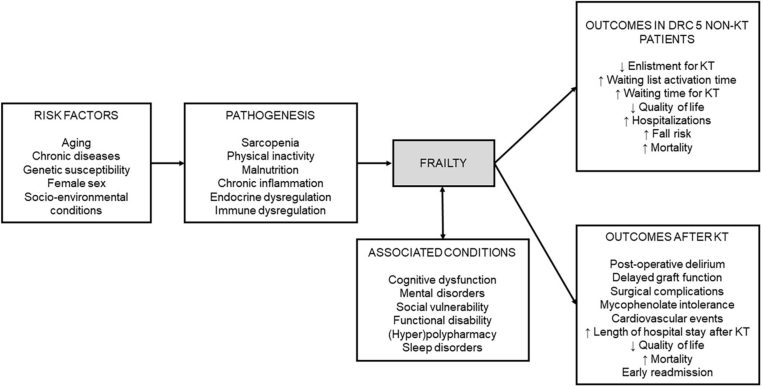
Risk factors, pathogenesis, associated conditions, and outcomes of frailty syndrome in the context of kidney transplantation.

Abbreviation: KT: kidney transplantation.

Figure 1 illustrates the multidimensional aspect of frailty syndrome and its associated conditions.

## Diagnosis

Several instruments have been developed aimed at diagnosing frailty syndrome. Some exclusively assess domains associated with physical frailty, while others also estimate domains that comprehensively encompass the state of vulnerability. Table 1 summarizes the most commonly described instruments in the literature. Most of them have already been tested on KT recipients^11,24,25,26,27,28,29,30,31,32,33,34,35,36,37,38,39,40,41,42,43^.

**Table 1.** Characteristics of the main tools used for diagnosing frailty

Tool	Author(s)	Domains Assessed	Advantages	Disadvantages
Instruments for Assessing the Physical Frailty Phenotype				
Fried Frailty Scale	Fried et al.^24^ (Cardiovascular Health Study, Johns Hopkins Medical Institutions – USA)	Muscle weakness (handgrip strength) Exhaustion Unintentional weight loss Physical inactivity Slow gait	Practical and easy to apply. Extensively validated in KT cohorts. Validated and/or cross-culturally adapted^25,26^.	Requires a dynamometer. Exhaustion may be subjective (self-reported). Physical inactivity may be subjective (self-reported). Limited for patients with motor deficits.
Short Physical Performance Battery (SPPB)	Welch et al.^27^(Vanderbilt University Medical Center – USA)	Slow gait Balance Muscle weakness (lower limbs)	Practical and easy to apply. Validated and/or cross-culturally adapted^28^.	Limited to patients with conditions affecting only lower limbs.
Instruments Based on the Accumulation of Deficits Model				
Edmonton Frail Scale	Rolfson et al.^29^ (University of Alberta – Canada)	Domains related to factors such as cognition, general health status, functional independence, and social support.	It is increasingly used in hospital practice, with adaptations for emergencies in the elderly^30^. Validated and/or cross-culturally adapted^31^.	It includes subjective questions. Assessment of complex domains such as cognition, depression, and physical performance in a single question may not accurately capture the patient’s condition.
Groningen Frailty Index	Steverink et al.^32^ (University of Groningen – Netherlands)	Domains related to mobility, vision, hearing, nutrition, comorbidities, cognition, psychosocial, and physical capacity.	Binary answers facilitate the test application. Widely used in the Netherlands, in conjunction with the Frailty Index. Validated and/or cross-culturally adapted^33^.	Binary answers may not properly capture the conditions. More extensive and complex, difficult to apply in the context of KT.
Tilburg Frailty Indicator	Gobbens et al.^34^ (Tilburg University – Netherlands)	Physical, psychological, and social domains through several questionnaires, such as: LASA Physical Activity Questionnaire, Timed Up & Go test, Loneliness Scale, and Social Support List, among others.	Physical components with good predictive value. Validated and/or cross-culturally adapted^35^.	Social components with low predictive value^34^. More extensive and complex, difficult to apply in the context of KT.
Kihon Checklist	Arai and Satake^36^(Care Prevention Programs – Japan)	Domains related to ADL, physical functionality, weight, appetite, mood, memory and willingness to perform activities through a questionnaire with 25 binary questions.	Binary answers facilitate the test application. Widely used in Japan. Validated and/or cross-culturally adapted^37^.	Binary answers may not properly capture the conditions.
Clinical Frailty Scale	Rockwood et al.^11^ (Geriatric Medicine Dalhousie University – Canada)	Assesses cognitive and physical domains, dependence on ADLs and IADLs, and the CSHA Frailty Index	Good outcome predictor in hospitalized elderly patients^38^. Validated and/or cross-culturally adapted^39^.	Being based on clinical judgment, it is subject to the evaluator’s subjectivity. More extensive and complex, difficult to apply in the context of KT.
Easycare Two-step Older Persons Screening (Easycare-TOS)	Van Kempen et al.^40^ (Radboud University Nijmegen Medical Centre – Netherlands)	14 domains: multimorbidity, polypharmacy, cognitive impairment, hearing and vision, ADL, mobility, falls, self-care, social support, depression, anxiety, somatic complaints, psychiatric complaints.	Used in primary care as a practical assessment tool.	Subjectivity of the evaluator, rather than a numerical score to certify frailty, increases variability. Two-stage assessment increases the need for time and personnel. Non-validated and/or lacking cross-cultural adaptation in Brazil. To date, there are no studies that have used this tool in KT recipients.
Clinical-Functional Vulnerability Index-20 (IVCF-20)	de Moraes et al.^41^ (Universidade Federal de Minas Gerais – Brazil)	8 domains: age, self-perceived health, functional disabilities, cognition, mood, mobility, communication, and multiple comorbidities.	Proposed as a tool for rapid screening of vulnerability in Brazilian elderly.	Applied and validated in elderly population and specialized hospital care. To date, there are no studies that have used this tool in KT recipients.
Canadian Study of Health and Aging (CSHA) Frailty Index	Mitnitski et al.^42^ (Ecole Polytechnique – Canada)	Domains related to 70 health deficits, including comorbidities, dependence on ADLs and IADLs, cognitive impairment, slow gait, depression, etc.	Flexible, due to the possibility of adapting the instrument based on the studied population. Good outcome predictor^43^.	Extensive and complex, difficult to apply in the context of KT. Calculations required. Non-validated and/or lacking cross-cultural adaptation in Brazil. To date, there are no studies that have used this tool in KT recipients.

Abbreviations: KT: Kidney Transplant; SPPB: Short Physical Performance Battery; CSHA: Canadian Study of Health and Aging; ADL: Activity of daily living; IADL: Instrumental activities of daily living.

Among them, the most widely used and validated in the context of CKD and KT is the Fried Physical Frailty Phenotype, which exclusively assesses physical reserve^24^. This instrument consists of assessing five domains: muscle weakness, assessed by handgrip strength; slow gait, assessed by walking speed; exhaustion, self-reported by the patient; unintentional weight loss, loss of 4 kg/year or more; and low physical activity, measured by the Minnesota Leisure Time Activity questionnaire^44^. The main advantage of this tool is the objectivity of the measures used to assess each domain, which minimizes inter- and intra-observer biases and variability. This favors its use not only for initial diagnosis but also as a follow-up tool. In addition, it has a strong capacity to predict outcomes, and is quite feasible to implement in daily clinical practice. The main disadvantages, given its purely phenotypic assessment proposal, are the need for a dynamometer and the inability to assess patients with lower limb deficits that compromise ambulation (Table 2)^24,44,45^.

**Table 2.** Fried frailty phenotype

**Weight loss**	The patient scores 1 point if they experienced unintentional weight loss of > 4.5 kg or > 5% of body mass in the past 12 months (self-reported)	
**Muscle weakness**	Using a dynamometer, 3 consecutive HGS measurements are taken. The patient scores 1 point if the average of the 3 values is below 20% of the expected value for sex and BMI, according to the values below:	
	**Men**	**Women**
	IMC ≤ 24 Kg/m^2^: HGS≤29kg	IMC ≤ 23 Kg/m^2^: HGS≤17kg
	IMC 24.1–26 Kg/m^2^: HGS≤30kg	IMC 23.1–26 Kg/m^2^: HGS≤17.3kg
	IMC 26.1–28 Kg/m^2^: HGS≤30kg	IMC 26.1–29 Kg/m^2^: HGS≤8kg
	IMC ≥ 28 Kg/m^2^: HGS≤32kg	IMC ≥ 29 Kg/m^2^: HGS≤21kg
**Exhaustion**	The patient is asked questions 7 and 20 from the Center for Epidemiological Studies Depression (CES-D) questionnaire:	
	*“How often in the past week did you feel that everything you did demanded a lot of effort?”*
	*“How often in the past week did you feel you could not get going?”*	
	The alternatives are as follows:	
	(0) Rarely or none of the time/1 day	
	(1) Some or a little of the time/1–2 days	
	(2) Occasionally or a moderate amount of the time/3–4 days	
	(3) Most or all of the time/always	
	The patient scores 1 point if they answer 2 or 3 to either of the two questions.	
**Slowness**	The walk test is performed, and the average of 3 consecutive measurements of the time taken to cover a distance of 4.6 meters is obtained. The patient scores (1 point) if their performance does not meet the minimum expected for their sex and height, as outlined below:	
	**Men**	**Women**
	Height ≤ 173cm: Time ≥ 7 sec	Height ≤ 159cm: Time ≥ 7 sec
	Height ≥ 173cm: Time ≥ 6 sec	Height ≥ 159cm: Time ≥ 7 sec
**Low physical activity**	The patient scores 1 point if their weekly energy expenditure was lower than 383 kcal for men and 270 kcal for women in the past two weeks, based on the short version of the Minnesota Leisure Time Activity44	
Once the points have been added up, the result is as follows*:		
	**0–1 point = non-frail**		
	**2 points: pre-frail**		
	**3–5 points = frail**		

Abbreviations: HGS: handgrip strength; BMI: body mass index.

Note: *The original classification by Fried et al.^24^ considers a score of 1 as pre-frail. However, in 2013, McAdams-DeMarco et al. proposed an adaptation for the CKD population, considering a score of 1 as non-frail^45^.

Recently, a group of researchers from Groningen and American universities proposed the Abridge Physical Frailty, a simplified version of the Physical Frailty Phenotype proposed by Fried and colleagues. This new tool retains the five domains, but tests them in a more optimized way, thus facilitating its implementation in daily routine. The validation study demonstrated that this new tool has good discriminating capacity and is associated with outcomes similar to those of the original tool, with application time reduced to approximately 10 minutes^46^.

Another interesting point would be to incorporate an objective parameter for assessing sarcopenia into the diagnostic tool, such as tomographic assessment of abdominal muscle groups or morphometric age, determined by aortic calcification and characteristics of the psoas muscle. Sarcopenia is a risk factor for mortality in patients on the KT waiting list^47^, and is directly involved in the pathogenesis of frailty^10^. Morphometric age has proven to be a predictor of both patient and graft survival in the short and long term^48^. However, further studies are needed to evaluate this instrument, and its implementation requires technologies that limit its use in routine practice.

## Epidemiology

The prevalence of frailty varies considerably across studies due to the diversity of instruments used and the demographic and clinical characteristics of each analyzed population. In the elderly population, the prevalence ranges from 4% to 59.1%, depending on the instrument used for assessment and the location of the study^49^. Considering the most commonly used instrument, proposed by Fried et al.^24^, the prevalence of frailty in the elderly is estimated to be between 4% and 17%^49^. Among CKD patients in stages 1 to 4, the observed prevalence ranges from 7% to 42.6%, increasing as the glomerular filtration rate decreases^12^. The only Brazilian study in the pre-dialysis CKD population, by Mansur et al., reports a prevalence of 42.6%, using the tool proposed by Fried et al.^50^.

Dialysis patients exhibit an even higher prevalence of frailty, ranging from 14% to 73%, and are affected at an earlier stage, with a prevalence of up to 63% in patients under the age of 40^12,51^. In the Brazilian population, Gesualdo et al. observed a prevalence of 47.7% of frail patients, and 44.9% of pre-frail or intermediate patients among a sample of 107 hemodialysis patients. Additionally, in this cohort, the likelihood of patients experiencing frailty increased by 3% for each additional year of life^52^. Another single-center Brazilian study found a higher prevalence of 73.8%^16^.

KT candidates represent a relatively healthier portion of the stage 5 CKD population, as patients with decompensated diseases and conditions that significantly compromise life expectancy are contraindicated for KT. Nevertheless, frailty syndrome is prevalent among patients on the waiting list, being reported in 13% to 18% of individuals^53,54,55^, which is quite similar in incident KT patients, ranging from 16% to 25%^56^. The only Brazilian study found a prevalence of 36.7% in a sample of 87 patients assessed at the time of KT, with a mean age of less than 50 years^57^.

After KT, frailty status varies considerably due to the complex interaction between immunosuppression and improved renal function. Recovery of physiological reserve is crucial both for improving frailty and for patient and graft survival. In a cohort of American patients, individuals became much frail one month after the procedure, an expected consequence of surgery and hospitalization. However, a progressive improvement subsequently occurs and, within months, an improvement in frailty status compared to the pre-transplant situation can already be observed. In this study, three months after kidney transplantation, only 25.9% of patients considered frail at the time of transplantation remained with the same diagnosis, 40.7% became pre-frail and 33.4% became non-frail^58^.

Despite this improvement observed in the first three months post-transplant, the frailty status varies considerably among different cohorts in the long term. In a prospective cohort study with a 5-year observation period involving 1336 KT recipients, a significant improvement in all domains of the Fried Frailty Phenotype was observed in the first 2.5 years post-KT, with the exception of slow gait. This effectively reduced the probability of being frail in this sample. However, this result was not sustained between 2.5 and 5 years post-KT, with stabilization and even worsening in some domains (handgrip strength), suggesting an increased likelihood of frailty in the long term^59^.

Conversely, Quint and colleagues did not observe the same improvement in their sample three years post-KT: approximately 20% of non-frail patients became frail during this period in a cohort of 233 patients in the Netherlands, using the Groningen Frailty Index tool^32^. This tool captures effects not observed by Fried’s tool, such as cognitive and psychosocial scores. If these are present at the time of transplantation, they increase the probability of the individual becoming frail post-KT^60^.

In a single-center Brazilian cohort of 64 KT recipients, patients became less frail one year after KT, with a 69.9% reduction (15.6% to 4.7%) in the number of frail individuals in the sample. A significant reduction was observed in the number of patients presenting with weight loss among the Fried Frailty Phenotype domains, from 34.4% at the time of KT to 6.3% one year after KT^61^.

These variations in the long-term evolution of frailty in KT patients are attributed to the multifaceted nature of frailty, the multiple tools available for its characterization, as well as the socioeconomic differences in the samples analyzed. In addition, factors associated with the KT population may have an impact on this evolution. These include the effects of chronic use of immunosuppressive medications^62^, the increased burden of chronic comorbidities, and the aging of patients themselves.

Table 3 summarizes the main studies that assessed the prevalence of frailty in CKD patients on the waiting list and at admission for KT^21,46,53,54,55,57,63,64,65,66,67,68,69,70,71,72,72,74,75,76,77,78,79,80,81,82,83,84,85,86,87,88,89,90,91^.

**Table 3.** Studies on the prevalence of frailty in waiting lists and at the time of kidney transplantation

First author	Year	Country	Sample size	Assessment tool	Age (years, mean ± SD or median, min-max)	Female (%)	Frailty (%)
Assessment Conducted in Patients on the Waiting List for KT							
Chen X^46^	2024	USA	220	FFP	–	–	23.8
Xu EJ^63^	2024	USA	101	SPPB and GFI	53.3 ± 12.0	35.6	39.6
Thind AK^64^	2023	England	186	EFS	66.0/65.1/64.6^a^	33.9/27.8/46.9^b^	17.2
Schaenman J^65^	2023	USA	514	SPPB/FFP	64 (55–84)	37.0	23.0/8.0^c^
1408	SPPB/FFP	64 (55–84)	37.0	18.0/6.0^c^
Pérez-Sáez MJ^66^	2022	Spain	296	FFP	62.6 ± 12.3	29.4	26.7
Pérez-Sáez MJ^67^	2022	Spain	451	FFP	60.9 ± 12.2	31.7	10.4
Chen X^68^	2022	USA	1113	FFP	52.9 ± 13.8	38.6	18.6
Pérez-Sáez MJ^21^	2021	Spain	455	FFP	60.6 ± 12.4	31.6	10.3
Haugen CE^69^	2021	USA	1154	FFP	54.0 ± 13.0	34.3	19.0
Worthen G^70^	2021	USA	542	FFP	54.0 ± 14.0	36.0	16.2
Chu NM^71^	2020	USA	4304	FFP	55.3 ± 14.8	40.8	12.3
Chu NM^72^	2020	USA	3666	FFP	54.0 ± 14.0	38.1	20.9
Haugen CE^73^	2020	USA	3143	FFP	54.0 ± 14.0	40.0	57.9
Pérez Fernandéz M^53^	2019	USA	2086	FFP	53.8 ± 13.5	40.1	18.1
Haugen CE^54^	2019	USA	4552	FFP	52.0 ± 13.0	61.0	12.3
Lorenz EC^74^	2019	USA	272	FFP	61.8 ± 9.3	37.9	14.3
Shrestha P^75^	2019	USA	1003	FFP	55.0 ± 13.0	40.0	19.1
Vera Casanova^76^	2017	Spain	177	FFP	62.1 ± 10.4	40.1	31.1^d^
Assessment Conducted at the Time of Admission for KT							
Parajuli S^77^	2022	USA	825	FFP	55.3^e^	40.0	11.5
Haugen CE^69^	2021	USA	378	FFP	54.0 ± 13.0	34.3	19.0
Dos Santos Mantovani M^57^	2020	Brazil	87	FFP	44.0 ± 12.0/ 46.0 ± 13.0^f^	41.4	16.1
Haugen CE^78^	2020	USA	4616	FFP	52.2 ± 13.4	45.7	13.3
Kosoku A^79^	2020	Japan	205	KCL	55.0 (45.0–65.0)	43.0	11.2
Thomas AG^80^	2020	USA	465	FFP	52.6 ± 15.6	37.2	13.3
Chu NM^55^	2019	USA	569	FFP	51.7 ± 14.0	39.2	16.0
Chu NM^81^	2019	USA	665	FFP	52.0 ± 14.2	38.8	15.0
Schopmeyer L^82^	2019	Netherlands	139	GFI	51.8 ± 14.5	37.4	16.5
Thomas AG^83^	2019	USA	864	FFP	53.0 ± 3.3	38.4	16.7
Haugen CE^84^	2018	USA	893	FFP	52.5 ± 14.2	39.0	16.4
Konel JM^85^	2018	USA	773	FFP	54.0 ± 14.0	37.8	16.3
McAdams-DeMarco MA^86^	2018	USA	443	FFP	52.0 ± 14.1	37.3	37.0^d^
Nastasi AJ^87^	2018	USA	719	FFP	51.6 ± 14.2	37.7	15.7
McAdams-DeMarco MA^88^	2017	USA	663	FFP	53.0 ± 13.9	38.0	19.5
McAdams-DeMarco MA^89^	2015	USA	537	FFP	53.0 ± 14.0	39.9	19.9
McAdams-DeMarco MA^62^	2015	USA	525	FFP	53.0 ± 14.0	39.8	19.5
McAdams-DeMarco MA^58^	2015	USA	349	FFP	53.3 ± 14.2	38.1	19.8
McAdams-DeMarco MA^90^	2013	USA	383	FFP	53.5 ± 13.9	39.7	18.8
Garonzik-Wang JM^91^	2012	USA	183	FFP	53.0 ± 14.0	36.0	25.1

Abbreviations: EFS: Edmonton Frail Scale; KT: kidney transplantation; FFP: Fried Frailty Phenotype; KCL: Kihon Checklist; GFI: Groningen Frailty Indicator; SPPB: Short Physical Performance Battery.

Notes: a. Reported as mean – non-frail/vulnerable/frail, b. Reported as percentage of non-frail/vulnerable/frail, c. SPPB/FFP, d. Includes intermediate and frail patients, e. Reported as mean, f. Reported as non-frail/frail patients.

## Impact on Outcomes

Frailty syndrome has been consistently associated with a higher risk of mortality and impaired quality of life across all CKD scenarios (pre-dialysis, on dialysis off the waiting list, patients on the waiting list for KT, and KT patients)^8,12,45,86,89^. In pre-dialysis patients, frailty is also associated with a greater risk of inability to perform daily activities, dependency, hospitalization, and falls^45,92^. Frail CKD patients are 30% to 38% less likely to be enlisted for KT. Once enlisted, they are 30% more likely to be inactivated and removed from the list. When on the list, there is a reported 70% higher risk of death and a 32% to 38% lower chance of being transplanted, compared to non-frail patients^54,56,93^ (Figure 1).

In the context of KT, the evidences are more heterogeneous, as studies are generally single-center, affecting the demographic profile studied, the diagnostic tool used, and the center’s policy on whether or not to transplant patients with high frailty scores. Considering some of the major evidence available in the literature, frailty has been linked to a number of early outcomes following KT. These include *delirium* (OR 2.05)^84^, delayed graft function (HR 1.78 to 1.80)^8,56^, surgical complications (HR 1.88)^8^, prolonged hospitalization (HR 1.55)^8^, and early readmission after discharge (HR 1.61)^77,90^.

Among the relevant late outcomes, patients with a frailty status at KT admission exhibit a nearly two-fold increase in the risk of all-cause mortality (HR 1.97). This risk remains elevated regardless of the diagnostic tool used or the length of follow-up periods, whether shorter or longer than 5 years^8^. It is worth noting that even in pre-frail patients, there is 1.5 times greater risk of post-KT death^89^. In contrast, an American retrospective cohort study of 19,242 dialysis patients, which assessed frailty using the physical component of the SF-36 (SF-12), also observed reduced patient survival (84% vs. 94%) when comparing the lowest quartile of values with the highest ones after KT. Nevertheless, for all four SF-12 quartiles, the benefit of enhanced survival was observed from the 9th month after KT when in comparison to persistence on dialysis. This suggests that the survival benefit following KT is present across the various stages of frailty^93^.

Increased intolerance to immunosuppressants, particularly mycophenolate^62^, as well as a higher incidence of cardiovascular events^77^ and impaired quality of life have also been reported^86^ (Figure 1).

## Interventions

Interventions to reduce frailty in populations with CKD, both pre- and post-KT, have not yet been properly studied. Experiences applied to the general population may, however, provide guidance on how to proceed. Due to its multifactorial nature, it is estimated that a combination of multiple actions could yield better results. Early measurement of results can be assessed through changes in the domains that are part of the diagnosis (such as muscle weakness, slowness and exhaustion). In the long term, this approach can prevent functional decline and impairment^9^. For patients on the waiting list, the interval until KT represents an opportunity for preventive intervention, as frailty status is dynamic^55^. Conversely, after KT, the measures could modify the undesirable outcomes associated with frailty in the medium and long term. The proposed measures include physical rehabilitation, nutritional supplementation, management of comorbidities, psychological support, and KT itself^9,94,95,96,97^.

Prior publications have reported the safety and feasibility of pre-rehabilitation interventions for individuals on the list for solid organ transplantation. These interventions resulted in improvements in cardiorespiratory function, exercise capacity, muscle strength, and quality of life^98,99^. However, there is no evidence regarding the best set of measures to be implemented^97^, with the exception of two institutional recommendations^97,100^. Regarding physical activity, the studies are limited to only three publications that used different types of exercise (yoga, resistance training, strength, and flexibility), over an 8-week training period, involving small samples. Nevertheless, improvements were observed in the frailty components, mental quality of life components and post-KT length of stay^101,102,103^.

Nutritional support, aimed at achieving optimal nutritional status with dietary recommendations from specialized professionals, is part of the treatment of CKD patients, regardless of whether or not they are on the transplant list. However, in frail patients, greater attention should be given to the presence of malnutrition. Interventions involving increased caloric intake and more frequent monitoring have been used in transplants of other organs^97^. Under the same rationale, optimizing hemoglobin levels would be justified by its association with inflammation and frailty in incident KT patients^104^. Better clinical control of comorbidities such as hypertension and diabetes mellitus would also be more relevant in this population^95,96^.

Pre-rehabilitation measures, therefore, require additional structuring of services. Even in the absence of evidence, it is estimated that these measures could increase patients’ motivation for KT, thereby serving as an emotional support measure^95^.

KT itself is probably the most effective strategy for improving the state of frailty. Even among the frailest patients, the benefit of reduced mortality after KT is observed^93^. Following surgical stress, there is an increased risk of developing frailty in the first month. However, from the third month onwards, there was an improvement in the domains comprising frailty and a significant reduction in the number of frail patients^58,60^. Specifically, improvements were noted in the domains of weight gain, muscle strength, and physical activity^59^. An improvement in uremia is probably the pivotal modifying factor. The prevalence and risk of becoming frail generally remain lower in the first year^60^, as reported in a Brazilian study by Dos Santos Mantovani et al.^61^ and, in the long term, after 2.5 years^59^. Nevertheless, due to its multifactorial nature, frailty status is dynamic after KT and in the long term. Especially after 2.5–3 years, there is a tendency for the strength and activity components to decline, which requires closer attention^59,60^. Given the available evidence, it can be considered that frailty status alone should not be regarded as a contraindication for KT^4,9,93^. In contrast, including it into the pre-KT assessment will enable a better approach both on the list and after KT with the aim of reducing events that increase morbidity. This is because subjective assessment without specific instruments could be inaccurate in up to 37% of cases^105^.

The same principles as for rehabilitation measures apply after KT. Programs aimed at increasing physical activity, despite not specifically evaluating the impact on frailty, have demonstrated efficacy in improving physical function and performance, which are components of the frailty phenotype. Additionally, they have enhanced quality of life and certain cardiovascular disease markers. The lack of robust evidence has prompted ongoing debate regarding which type of exercise, intensity, frequency, and duration would provide the greatest benefit^106^. It is recommended to include aerobic exercises, whether or not combined with resistance exercises, of moderate to high intensity, 3–5 times a week, for at least 8 weeks^100^.

## Final Messages

Frailty syndrome is a common and underdiagnosed condition in patients with stage 5 CKD who are candidates for KT. Underdiagnosis results from the uncertainty about the optimal tool for diagnosis and, notably, from the difficulty of operationalizing this assessment on a routine and periodic basis. Frailty, which has a multifactorial etiology, encompasses multidimensional components that extend beyond the physical phenotype, implying a broader status of vulnerability. This negatively impacts access to the waiting list and KT itself, as well as early and late outcomes following KT. The need to establish a routine for the diagnosis, management and follow-up of frailty before and after KT is urgent, aiming at improving patient outcomes^4^.
