# Supplementary material for: Frailty in the context of kidney transplantation
Source: J Bras Nefrol. 2024 Sep 27;46(4):e20240048. doi: 10.1590/2175-8239-JBN-2024-0048en (PMC11435774; doi:10.1590/2175-8239-JBN-2024-0048en)
Supplement: Translation

# Translation: Fragilidade no contexto do transplante renal

**DOI:** 10.1590/2175-8239-JBN-46-4-E20240048pt

**Authors:** Tainá Veras de Sandes-Freitas, Raoni de Oliveira Domingues-da-Silva, Helady Sanders-Pinheiro

**Affiliations:** 1 Universidade Federal do Ceará, Faculdade de Medicina, Fortaleza, CE, Brasil.; 2 Hospital Geral de Fortaleza, Fortaleza, CE, Brasil.; 3 Universidade Federal de Juiz de Fora, Faculdade de Medicina, Núcleo Interdisciplinar de Estudos e Pesquisas em Nefrologia (NIEPEN), Juiz de Fora, MG, Brasil.; 4 Universidade Federal de Juiz de Fora, Hospital Universitário, Serviço de Transplante Renal, Juiz de Fora, MG, Brasil.

**Keywords:** Fragilidade, Transplante Renal, Doença Renal Crônica, Incidência, Fatores de Risco, Acesso aos Serviços de Saúde

## Abstract

A fragilidade, conceituada como a resposta inadequada a situações de estresse devido à perda da reserva fisiológica, foi descrita incialmente na população idosa, mas atualmente vem sendo identificada em populações mais jovens com doenças crônicas, como a doença renal crônica. Estima-se que cerca de 20% dos pacientes sejam frágeis no momento do transplante renal (TR), e há grande interesse pelo seu potencial valor preditor de desfechos desfavoráveis. Um número expressivo de evidências tem sido gerado, entretanto, ainda persistem várias áreas a serem mais exploradas. A patogênese é pouco conhecida e limitada à extrapolação dos achados de outras populações. A maioria dos estudos é observacional, envolvendo pacientes em lista ou após o TR, e há escassez de dados sobre a evolução em longo prazo e possíveis intervenções. Revisamos os estudos, incluindo aqueles com populações brasileiras, de avaliação de fragilidade nas fases pré e pós-TR, explorando a fisiopatologia, os fatores associados, os desafios do diagnóstico e os desfechos associados, na tentativa de fornecer embasamento para futuras intervenções.

## Introdução

Com os avanços na eficiência dos imunossupressores, na seleção de receptores (técnicas de tipagem HLA e identificação de anticorpos específicos contra o doador) e melhor manuseio de infecções, a sobrevida em longo prazo dos enxertos renais tem melhorado significativamente. Essa melhora, no entanto, não é uniforme para todos os grupos de pacientes^1^. Entre as potenciais explicações está a mudança do perfil epidemiológico dos candidatos ao transplante renal (TR). Mesmo que os pacientes em lista para TR sejam os mais saudáveis, a média de idade dos pacientes aumentou, assim como as comorbidades^2,3^. Uma maneira de avaliar de forma mais fidedigna as condições clínicas no momento do TR é identificando a presença de fragilidade^4^. Inicialmente desenvolvido como um instrumento para apropriadamente se distinguir a idade fisiológica da cronológica em indivíduos mais velhos, o diagnóstico da síndrome de fragilidade tem sido utilizado para identificar mais acuradamente a vulnerabilidade a desfechos indesejados para além da idade ou presença de morbidades. Mais recentemente, tem sido identificada e estudada em populações não idosas, como em pacientes com doença renal crônica (DRC) e TR^4,5^.

Fragilidade é uma entidade que se caracteriza pela resposta inadequada às situações de estresse pela perda da reserva fisiológica^6^. Embora se sobreponha à presença de morbidades e limitação física, é fator de risco independente para piores desfechos em pacientes com DRC e TR^7,8^. Sua identificação e de fatores associados potenciais pode promover abordagem precoce tanto para o desenvolvimento de medidas preventivas no manuseio de candidatos quanto após o TR^4,9^.

Realizamos revisão nas bases SciELO, PubMed e LILACS usando os seguintes termos: “frailty” e “kidney transplantation”, no período de 2012 a 2024. Foi feita também busca secundária a partir de artigos selecionados pela busca principal que se adequavam aos objetivos do artigo.

## Definição, Patogenia, Fatores de Risco e Condições Associadas

A fragilidade é uma condição de etiologia multifatorial resultante da desregulação ou deterioração da homeostase e das reservas fisiológicas e maior vulnerabilidade aos estressores ambientais e internos. A síndrome de fragilidade pode ser entendida como uma condição puramente física (fenótipo de fragilidade física) ou como uma síndrome multidimensional de vulnerabilidade (acúmulo de déficits)^6^.

Entre os componentes fundamentais do fenótipo de fragilidade física, destaca-se a sarcopenia, que se caracteriza pela redução da massa, força e função muscular, comumente associada ao envelhecimento. No entanto, a sarcopenia isoladamente não explica a fragilidade, e essas condições não devem ser entendidas como sinônimos, apesar de frequentemente estarem associadas. Outros fatores além da sarcopenia compõem a patogenia da síndrome de fragilidade física, como inatividade, desnutrição, estados inflamatórios crônicos, desregulação hormonal e do sistema imunológico, entre outras condições clínicas^10^. Para além do componente físico, um indivíduo pode ser compreendido como frágil por apresentar outras vulnerabilidades, como deterioração cognitiva e comprometimento de componentes do domínio psicológico, como depressão, e do domínio social, como baixa escolaridade, baixa renda e falta de rede de apoio^11^.

A síndrome de fragilidade foi descrita inicialmente no contexto do envelhecimento e do consequente comprometimento das funções sistêmicas. De fato, essa é uma condição mais prevalente entre idosos, entretanto, pode ser observada também em indivíduos não idosos portadores de determinadas condições clínicas, notadamente doenças crônico-degenerativas, como a DRC. O mecanismo pelo qual a DRC está associada à fragilidade, independentemente da idade, não está completamente esclarecido, mas é provável que inflamação crônica, anemia, desnutrição, sarcopenia e inatividade estejam implicados na patogênese^12^.

Além do envelhecimento e de doenças crônicas, a suscetibilidade genética e as condições socioambientais desempenham um papel significativo na determinação da fragilidade. Entre os fatores genéticos, destacam-se o reparo do DNA e a redução da velocidade de erosão do telômero, funções fundamentais desempenhadas pelos complexos de proteínas p53 e p16. Estudos indicam que a proteína p53 está associada à idade, como evidenciado por pesquisas de polimorfismo em homens e mulheres^13^. Além disso, a superexpressão da proteína p16 em ratos demonstrou ter um efeito antienvelhecimento, sugerindo uma associação entre os genes de proteção e reparo do DNA e a fragilidade^13^. Por outro lado, Kumar e cols.^14^ identificaram uma associação significativa entre baixos níveis de sirtuínas, uma família de proteínas com atividade enzimática de desacetilação, e fragilidade.

Quanto às condições socioambientais, o acesso limitado a recursos de saúde e suporte social inadequado podem aumentar a vulnerabilidade à fragilidade, independentemente da faixa etária. Em um estudo transversal com 727 mulheres, observou-se que mulheres com nível educacional até o ensino fundamental tinham probabilidade até três vezes maior de serem frágeis do que indivíduos com mais educação, independentemente de idade, raça ou seguro de saúde^15^.

Outras condições comumente associadas à síndrome de fragilidade são transtornos mentais, como depressão, disfunção cognitiva, distúrbios do sono e perda da capacidade funcional para execução das atividades básicas e instrumentais de vida diária^16,17,18,19^. Além disso, polifarmácia (uso regular de ≥ 5 medicações) e hiperpolifarmácia (≥ 10 medicações) têm sido descritas nesse grupo de pacientes frágeis, inclusive entre transplantados renais^20^.

O sexo biológico também é um fator relevante na avaliação do fenótipo de fragilidade em candidatos a TR, sendo a prevalência dessa condição até duas vezes maior no sexo feminino^21^. Os mecanismos responsáveis pelo maior risco de fragilidade entre mulheres ainda precisam ser mais esclarecidos. Curiosamente, apesar de serem mais frequentemente diagnosticadas como frágeis, mulheres com DRC em tratamento conservador apresentam menor mortalidade que os homens, o que é conhecido como o “paradoxo dos sexos”, efeito não observado quando considerados os pacientes em diálise e após o TR^22,23^.

A Figura 1 ilustra o aspecto multidimensional da síndrome de fragilidade e as condições a ela associadas.

**Figura 1.** Fatores de risco, patogenia, condições associadas e desfechos da síndrome de fragilidade no contexto do transplante renal.

Abreviação: TR: transplante renal.

## Diagnóstico

Diversos instrumentos foram desenvolvidos com o intuito de diagnosticar a síndrome de fragilidade. Alguns avaliam exclusivamente domínios associados à fragilidade física, outros estimam também domínios que compõem o estado de vulnerabilidade de forma mais abrangente. A Tabela 1 resume os instrumentos mais comumente descritos na literatura, a maioria dos quais já foi testada em receptores de TR^11,24,25,26,27,28,29,30,31,32,33,34,35,36,37,38,39,40,41,42,43^.

**Tabela 1.** Características das principais ferramentas utilizadas para o diagnóstico de fragilidade

Ferramenta	Autor(es)	Domínios Avaliados	Vantagens	Desvantagens
Instrumentos Que Avaliam o Fenótipo Físico de Fragilidade				
*Fried Frailty Scale*	Fried et al.^24^ (*Cardiovascular Health Study,* Johns Hopkins Medical Institutions *–* EUA)	Fraqueza muscular (preensão de palmar) Exaustão Perda de peso não intencional Inatividade física Lentidão de marcha	Prático e de fácil aplicação. Extensamente validado em coortes de TR. Validada e/ou com adaptação transcultural^25,26^.	Necessidade de dinamômetro. Exaustão pode ser subjetiva (autorrelato). Inatividade física pode ser subjetiva (autorrelato). Limitado para pacientes com déficits motores.
*Short Physical Performance Battery* (SPPB)	Welch et al.^27^ (Vanderbilt University Medical Center – EUA)	Lentidão de marcha Equilíbrio Fraqueza muscular (membros inferiores)	Prático e de fácil aplicação Validado e/ou com adaptação transcultural^28^.	Limitado para pacientes com condições que afetem membros inferiores apenas.
Instrumentos Que se Baseiam no Modelo de Acúmulo de Déficits				
*Edmonton Frail Scale*	Rolfson et al.^29^ (University of Alberta – Canadá)	Domínios relacionados a fatores como cognição, estado geral de saúde, independência funcional, suporte social.	Vem sendo utilizado cada vez mais na prática hospitalar, com adaptações para emergências em idosos^30^. Validado e/ou com adaptação transcultural^31^.	Possui quesitos subjetivos. Avaliação de domínios complexos, como cognição, depressão e *performance* física em apenas uma pergunta pode não captar corretamente o estado do paciente.
*Groningen Frailty Index*	Steverink et al.^32^ (*University of Groningen* – Holanda)	Domínios relacionados a mobilidade, visão, audição, nutrição, comorbidades, cognição, psicossocial e capacidade física.	Respostas binárias facilitam a aplicação do teste. Amplamente utilizado na Holanda, em conjunto com o *Frailty Index.* Validado e/ou com adaptação transcultural^33^.	Respostas binárias podem não capturar de maneira adequada as condições. Mais longo e complexo, de difícil aplicação no contexto do TR.
*Tilburg Frailty Indicator*	Gobbens et al.^34^(Tilburg University – Holanda)	Domínios físicos, psicológicos e sociais por meio de vários questionários, tais como: LASA *Physical Activity Questionnaire, Timed Up & Go test, Loneliness Scale,* e *Social Support List,* entre outras.	Componentes físicos com bom valor preditivo. Validado e/ou com adaptação transcultural^35^.	Componentes sociais com baixo valor preditivo^34^. Mais longo e complexo, de difícil aplicação no contexto do TR.
*Kihon Checklist*	Arai e Satake^36^(*Care Prevention Programs* – Japão)	Domínios relacionados a AVD, funcionalidade física, peso, apetite, humor, memória e disposição para atividades por meio de um questionário com 25 questões binárias.	Respostas binárias facilitam a aplicação do teste. Amplamente utilizado no Japão. Validado e/ou com adaptação transcultural^37^.	Respostas binárias podem não capturar de maneira adequada as condições.
*Clinical Frailty Scale*	Rockwood et al.^11^ (*Geriatric Medicine Dalhousie University* – Canada)	Avalia domínios cognitivos e físicos, dependência em AVD e AIVD e CSHA *Frailty Index*	Bom preditor de desfechos em idosos hospitalizados^38^. Validado e/ou com adaptação transcultural^39^.	Por ser baseado no julgamento clínico, está sujeito à subjetividade do avaliador. Mais longo e complexo, de difícil aplicação no contexto do TR.
*Easycare Two-step Older Persons Screening* (Easycare-TOS)	Van Kempen et al.^40^(Radboud University Nijmegen Medical Centre – Holanda)	14 domínios: multimorbidade, polifarmácia, déficit cognitivo, escuta e visão, AVD, mobilidade, quedas, autocuidado, suporte social, depressão, ansiedade, queixas somáticas, queixas psiquiátricas.	Utilizado na atenção primária como ferramenta prática de avaliação.	Subjetividade do avaliador ao invés de uma pontuação numérica para atestar fragilidade aumenta a variabilidade. Avaliação em duas etapas aumenta necessidade de tempo e equipe. Não validado e/ou sem adaptação transcultural no Brasil. Até o momento, não há estudos que utilizaram esta ferramenta em receptores de TR.
*Clinical-Functional Vulnerability Index-20* (IVCF-20)	de Moraes et al.^41^ (Universidade Federal de Minas Gerais – Brasil)	8 domínios: idade, autopercepção da saúde, incapacidades funcionais, cognição, humor, mobilidade, comunicação e múltiplas comorbidades.	Proposto para ser um instrumento de triagem rápida de vulnerabilidade em idosos brasileiros.	Aplicado e validado em população idosa e em atendimento hospitalar especializado. Até o momento, não há estudos que utilizaram esta ferramenta em receptores de TR.
*Canadian Study of Health and Aging (CSHA) Frailty Index*	Mitnitski et al.^42^ (Ecole Polytechnique – Canadá)	Domínios relacionados a 70 déficits de saúde, incluindo comorbidades, dependência em AVD e AIVD, comprometimento cognitivo, lentidão de marcha, depressão, etc.	Flexível, pela possibilidade de adaptar o instrumento a partir da população estudada. Bom preditor de desfechos^43^.	Longo e complexo, de difícil aplicação no contexto do TR. Necessidade de cálculos. Não validado e/ou sem adaptação transcultural no Brasil. Até o momento, não há estudos que utilizaram esta ferramenta em receptores de TR.

Abreviações: TR: Transplante Renal; SPPB: *Short Physical Performance Battery*; CSHA: *Canadian Study of Health and Aging*; AVD: Atividade de vida diária; AIVD: Atividade instrumental de vida diária.

Entre eles, o mais utilizado e validado no contexto da DRC e do TR é o Fenótipo de Fragilidade Física de Fried (*Physical Frailty Phenotype*), que avalia exclusivamente a reserva física^24^. Esse instrumento consiste na avaliação de cinco domínios: fraqueza muscular, avaliada pela preensão palmar; lentidão de marcha, avaliada pela velocidade de caminhada; exaustão, autorreferida pelo paciente; perda de peso não intencional, perda de 4 kg/ano ou mais; e baixa atividade física, mensurado pelo questionário *Minnesota Leisure Time Activity* ^44^. A principal vantagem dessa ferramenta é a objetividade das medidas utilizadas na avaliação de cada domínio, o que minimiza vieses e variabilidades inter e intra-observador, favorecendo sua utilização como um instrumento para seguimento, além de diagnóstico inicial. Além disso, ele tem boa capacidade de predizer desfechos e é bastante factível de ser implementado da prática clínica diária. As principais desvantagens, considerando a sua proposta de avaliação fenotípica exclusivamente, são a necessidade de dinamômetro e a impossibilidade de avaliar pacientes com déficits de membros inferiores que comprometam a deambulação (Tabela 2)^24,44,45^.

**Tabela 2.** Fenótipo de fragilidade de fried

**Perda de peso**	O paciente ganha 1 ponto caso apresente perda de peso não intencional de > 4,5 kg ou > 5% de massa corporal nos últimos 12 meses (autorreferida)		
**Fraqueza muscular**	Utilizando um dinamômetro, são realizadas 3 medidas consecutivas da FPM. O paciente ganha 1 ponto se a média dos 3 valores for abaixo de 20% do esperado para o sexo e IMC, de acordo com os valores abaixo:		
	**Homens**	**Mulheres**
	IMC ≤ 24 Kg/m^2^: FPM≤29kg	IMC ≤ 23 Kg/m^2^: FPM≤17kg
	IMC 24,1–26 Kg/m^2^: FPM≤30kg	IMC 23,1–26,0Kg/m^2^: FPM≤17,3kg
	IMC 26,1–28 Kg/m^2^: FPM≤30kg	IMC 26,1–29 Kg/m^2^: FPM≤18kg
	IMC ≥ 28 Kg/m^2^: FPM≤32kg	IMC ≥ 29 Kg/m^2^: FPM≤21kg
**Exaustão**	São feitas ao paciente as perguntas 7 e 20 do questionário *Center for Epidemiological Studies Depression* (CES-D):	
	*“Com que frequência na última semana sentiu que tudo o que fez exigiu um grande esforço?”*	
	*“Com que frequência na última semana sentiu que não conseguiria levar suas coisas adiante?”*	
	As alternativas são as que seguem:	
	(0) Raramente/1 dia	
	(1) Poucas vezes/1–2 dias	
	(2) Muitas vezes/3–4 dias	
	(3) Na maior parte do tempo/sempre	
	O paciente ganha 1 ponto caso responda 2 ou 3 em uma das duas questões	
**Lentidão**	É realizado o teste da caminhada, sendo obtida a média de 3 medidas consecutivas do tempo gasto para percorrer uma distância de 4,6 metros. O paciente pontua (1 ponto) caso não tenha o desempenho mínimo esperado para o sexo e a altura, conforme abaixo:	
	**Homens**	**Mulheres**
	Altura ≤ 173cm: Tempo ≥ 7 seg	Altura ≤ 159cm: Tempo ≥ 7 seg
	Altura ≥ 173cm: Tempo ≥ 6 seg	Altura ≥ 159cm: Tempo ≥ 7 seg
**Baixa atividade física**	O paciente ganha 1 ponto caso tenha um gasto calórico semanal inferior a 383 kcal para homens e 270 kcal para mulheres nas duas últimas semanas, com base na versão curta do *Minnesotta Leisure Time Activity* ^44^	
Após a somatória dos pontos, o resultado segue conforme abaixo*:		
	**0–1 ponto = não frágil**	
	**2 pontos: pré-frágil**	
	**3–5 pontos = frágil**	

Abreviações: FPM: força de preensão manual; IMC: índice de massa corporal.

Nota: *A classificação original de Fried e colaboradores^24^ considera 1 como pré-frágil. Entretanto, McAdams-DeMarco e colaboradores propuseram em 2013 uma adaptação para a população com DRC, considerando 1 como não frágil^45^.

Recentemente, um grupo de pesquisadores de universidades americanas e de Groningen propuseram a *Abridge Physical Frailty*, uma simplificação da *Physical Frailty Phenotype* proposta por Fried e colaboradores, mantendo os cinco domínios, mas testando-os de forma mais otimizada, facilitando sua implementação na rotina diária. O estudo de validação demonstrou que essa nova ferramenta tem boa capacidade discriminante e associação com desfechos similar à da ferramenta original, com um tempo de aplicação reduzido para em torno de 10 minutos^46^.

Outro ponto interessante seria a incorporação ao instrumento diagnóstico de parâmetro objetivo da avaliação de sarcopenia, como, por exemplo, avaliação tomográfica de grupos musculares abdominais ou da idade morfométrica, acessada por calcificação aórtica e caraterísticas do músculo psoas. A sarcopenia é fator de risco de mortalidade para pacientes em lista de espera para TR^47^ e está diretamente envolvida na patogenia da fragilidade^10^. A idade morfométrica tem se mostrado preditora de sobrevida do paciente e enxerto em curto e longo prazo^48^. Entretanto, estudos adicionais para avaliação desse instrumento são necessários e sua implementação requer tecnologias que limitam seu uso na prática rotineira.

## Epidemiologia

A prevalência de fragilidade varia consideravelmente entre estudos devido à diversidade de instrumentos utilizados e às características demográficas e clínicas de cada população analisada. Na população de idosos, essa variação fica entre 4% a 59,1%, a depender do instrumento utilizado para a avaliação e da localização do estudo^49^. Considerando-se o instrumento mais utilizado, proposto por Fried et al.^24^, a prevalência de fragilidade em idosos é estimada entre 4 a 17%^49^. Entre os pacientes com DRC nos estágios 1 a 4, a prevalência observada varia entre 7 e 42,6%, aumentando à medida que a taxa de filtração glomerular diminui^12^. O único estudo brasileiro na população com DRC pré-diálise, de Mansur e cols., relata prevalência de 42,6%, utilizando o instrumento de Fried et al.^50^.

Pacientes em diálise apresentam prevalência ainda maior de fragilidade, variando entre 14% e 73%, e são afetados de forma mais precoce, com uma prevalência de até 63% em pacientes com menos de 40 anos^12,51^. Na população brasileira, Gesualdo e cols. observaram, em uma amostra de 107 pacientes em hemodiálise, prevalência de 47,7% de pacientes frágeis e 44,9% de pré-frágeis ou intermediários. Além disso, nessa coorte, a chance de os pacientes apresentarem fragilidade aumentou em 3% a cada ano de vida^52^. Prevalência mais elevada, de 73,8%, foi encontrada em outro estudo brasileiro de centro único^16^.

Candidatos a transplante renal representam uma parcela relativamente mais saudável da população com DRC estágio 5, pois pacientes com doenças descompensadas e condições que comprometam de forma significativa a expectativa de vida são contraindicados para o TR. Ainda assim, a síndrome de fragilidade é prevalente entre pacientes em lista de espera, sendo reportada em 13 a 18% dos indivíduos^53,54,55^, bem semelhante em pacientes incidentes em TR, que varia de 16 a 25%^56^. O único estudo brasileiro encontrou prevalência de 36,7%, em amostra de 87 pacientes avaliados no momento do TR, com média de idade inferior a 50 anos^57^.

Após o TR, o *status* de fragilidade varia consideravelmente devido à interação complexa entre a supressão imunológica e a melhora da função renal, sendo a recuperação da reserva fisiológica fundamental tanto para a melhora da fragilidade quanto para a sobrevida do paciente e do enxerto. Em uma coorte de pacientes americanos, os pacientes tornam-se mais frágeis na avaliação realizada 1 mês após o procedimento, uma consequência esperada da cirurgia e hospitalização. Entretanto, uma melhora progressiva ocorre subsequentemente e, em meses, já pode ser observada melhora do status de fragilidade em relação à situação pré-transplante. Neste estudo, 3 meses após o transplante renal, apenas 25,9% dos pacientes frágeis no momento do transplante permaneceram com o mesmo diagnóstico, 40,7% tornaram-se pré-frágeis e 33,4% tornaram-se não frágeis^58^.

Não obstante essa melhora observada nos 3 primeiros meses pós-transplante, o *status* de fragilidade varia consideravelmente entre as diferentes coortes em longo prazo. Em estudo de coorte prospectiva com observação de 5 anos, numa amostra de 1336 receptores de TR, foi observada melhora significativa em todos os domínios do Fenótipo de Fragilidade de Fried nos 2,5 primeiros anos pós-TR, exceto lentidão de marcha, efetivamente reduzindo a probabilidade de ser frágil naquela amostra. Esse resultado, entretanto, não se sustentou entre 2,5 e 5 anos pós-TR, observando-se uma estabilização e até piora em alguns domínios (preensão palmar), o que sugere aumento na probabilidade de ser frágil em longo prazo^59^.

Por outro lado, Quint e colaboradores não observaram a mesma melhora em sua amostra 3 anos pós-TR: aproximadamente 20% dos pacientes não frágeis se tornaram frágeis nesse período em uma coorte de 233 pacientes na Holanda, utilizando a ferramenta *Groningen Frailty Index* ^32^. Essa ferramenta capta efeitos não observados pela ferramenta de Fried, tais como os escores de cognição e psicossocial, que, caso estejam presentes no momento do transplante, aumentam a probabilidade de o indivíduo tornar-se frágil pós-TR^60^.

Em uma coorte unicêntrica brasileira incluindo 64 receptores de TR, os pacientes tornaram-se menos frágeis um ano após o TR, com redução de 69,9% (15,6% para 4,7%) no número de indivíduos frágeis da amostra. Dentre os domínios do Fenótipo de Fragilidade de Fried, houve redução significativa do número de pacientes com perda de peso, de 34,4% no momento do TR para 6,3% um ano pós-TR^61^.

Essas variações na evolução de longo prazo da fragilidade nos pacientes transplantados renais devem-se ao caráter multifacetado da condição de fragilidade, às múltiplas ferramentas disponíveis para sua caracterização, bem como às diferenças socioeconômicas das amostras analisadas. Além disso, fatores associados à população de TR podem impactar essa evolução, tais como: o impacto do uso crônico de medicações imunossupressoras^62^, o aumento da carga de comorbidades crônicas e o próprio envelhecimento do paciente.

A Tabela 3 resume os principais estudos que avaliaram a prevalência de fragilidade em pacientes com DRC em lista de espera e na admissão para o TR^21,46,53,54,55,57,63 64,65,66,67,68,69,70,71,72,72,74,75,76,77,78,79,80,81,82,83,84,85,86,87,88,89,90,91^.

**Tabela 3.** Estudos de prevalência de fragilidade em lista de espera e no momento do transplante renal

Primeiro autor	Ano	País	Tamanho da amostra	Ferramenta de avaliação	Idade (anos, média ± DP ou mediana, mín.-máx.)	Feminino (%)	Fragilidade (%)
Avaliação foi Realizada em Pacientes em Lista de Espera Para TR							
Chen X^46^	2024	EUA	220	FFP	–	–	23,8
Xu EJ^63^	2024	EUA	101	SPPB e GRI	53,3 ± 12,0	35,6	39,6
Thind AK^64^	2023	Inglaterra	186	EFS	66,0/65,1/64,6^a^	33,9/27,8/46,9^b^	17,2
Schaenman J^65^	2023	EUA	514	SPPB/FFP	64 (55–84)	37,0	23,0/8,0^c^
1408	SPPB/FFP	64 (55–84)	37,0	18,0/6,0^c^
Pérez-Sáez MJ^66^	2022	Espanha	296	FFP	62,6 ± 12,3	29,4	26,7
Pérez-Sáez MJ^67^	2022	Espanha	451	FFP	60,9 ± 12,2	31,7	10,4
Chen X^68^	2022	EUA	1113	FFP	52,9 ± 13,8	38,6	18,6
Pérez-Sáez MJ^21^	2021	Espanha	455	FFP	60,6 ± 12,4	31,6	10,3
Haugen CE^69^	2021	EUA	1154	FFP	54,0 ± 13,0	34,3	19,0
Worthen G^70^	2021	EUA	542	FFP	54,0 ± 14,0	36,0	16,2
Chu NM^71^	2020	EUA	4304	FFP	55,3 ± 14,8	40,8	12,3
Chu NM^72^	2020	EUA	3666	FFP	54,0 ± 14,0	38,1	20,9
Haugen CE^73^	2020	EUA	3143	FFP	54,0 ± 14,0	40,0	57,9
Pérez Fernandéz M^53^	2019	EUA	2086	FFP	53,8 ± 13,5	40,1	18,1
Haugen CE^54^	2019	EUA	4552	FFP	52,0 ± 13,0	61,0	12,3
Lorenz EC^74^	2019	EUA	272	FFP	61,8 ± 9,3	37,9	14,3
Shrestha P^75^	2019	EUA	1003	FFP	55,0 ± 13,0	40,0	19,1
Vera Casanova^76^	2017	Espanha	177	FFP	62,1 ± 10,4	40,1	31,1^d^
Avaliação foi Realizada no Momento da Admissão Para O TR							
Parajuli S^77^	2022	EUA	825	FFP	55,3^e^	40,0	11,5
Haugen CE^69^	2021	EUA	378	FFP	54,0 ± 13,0	34,3	19,0
Dos Santos Mantovani M^57^	2020	Brasil	87	FFP	44,0 ± 12,0/ 46,0 ± 13,0^f^	41,4	16,1
Haugen CE^78^	2020	EUA	4616	FFP	52,2 ± 13,4	45,7	13,3
Kosoku A^79^	2020	Japão	205	KCL	55,0 (45,0–65,0)	43,0	11,2
Thomas AG^80^	2020	EUA	465	FFP	52,6 ± 15,6	37,2	13,3
Chu NM^55^	2019	EUA	569	FFP	51,7 ± 14,0	39,2	16,0
Chu NM^81^	2019	EUA	665	FFP	52,0 ± 14,2	38,8	15,0
Schopmeyer L^82^	2019	Holanda	139	GFI	51,8 ± 14,5	37,4	16,5
Thomas AG^83^	2019	EUA	864	FFP	53,0 ± 3,3	38,4	16,7
Haugen CE^84^	2018	EUA	893	FFP	52,5 ± 14,2	39,0	16,4
Konel JM^85^	2018	EUA	773	FFP	54,0 ± 14,0	37,8	16,3
McAdams-DeMarco MA^86^	2018	EUA	443	FFP	52,0 ± 14,1	37,3	37,0^d^
Nastasi AJ^87^	2018	EUA	719	FFP	51,6 ± 14,2	37,7	15,7
McAdams-DeMarco MA^88^	2017	EUA	663	FFP	53,0 ± 13,9	38,0	19,5
McAdams-DeMarco MA^89^	2015	EUA	537	FFP	53,0 ± 14,0	39,9	19,9
McAdams-DeMarco MA^62^	2015	EUA	525	FFP	53,0 ± 14,0	39,8	19,5
McAdams-DeMarco MA^58^	2015	EUA	349	FFP	53,3 ± 14,2	38,1	19,8
McAdams-DeMarco MA^90^	2013	EUA	383	FFP	53,5 ± 13,9	39,7	18,8
Garonzik-Wang JM^91^	2012	EUA	183	FFP	53,0 ± 14,0	36,0	25,1

Abreviações: EFS: Edmonton Frail Scale; TR: transplante renal; FFP: Fenótipo de Fragilidade de Fried; KCL: Escala de Kihon; GFI: Indicador de fragilidade de Groningen; SPPB: *Short Physical Performance Battery*.

Notas: a. Reportado como média – não frágeis/vulneráveis/frágeis, b. Reportado como percentual de não frágeis/vulneráveis/frágeis, c. SPPB/FFP, d. Incluídos pacientes intermediários e frágeis, e. Reportado como média, f. Reportado como pacientes não frágeis/frágeis.

## Impacto nos Desfechos

A síndrome de fragilidade tem sido consistentemente associada a maior risco de mortalidade e comprometimento da qualidade de vida em todas os cenários da DRC (pré-diálise, em diálise fora de lista, pacientes em lista de espera para o TR e transplantados renais)^8,12,45,86,89^. Naqueles em pré-diálise, a fragilidade está associada também a maior risco de incapacidade para as atividades diárias, dependência, hospitalização e quedas^45,92^. Pacientes frágeis com DRC têm de 30% a 38% menos chance de ser alistados para o TR e, uma vez listados, apresentam 30% mais chance de ser inativados e retirados da lista. Uma vez em lista, é reportado risco 70% maior de óbito e 32% a 38% menos chance de serem transplantados, comparados com os pacientes não frágeis^54,56,93^ (A Figura 1).

No contexto do TR, as evidências são mais heterogêneas, pois os estudos são geralmente unicêntricos, o que impacta a demografia estudada, a ferramenta utilizada para o diagnóstico e a política do centro de transplantar ou não pacientes com elevados escores de fragilidade. Considerando algumas das principais evidências disponíveis na literatura, foi relatada associação de fragilidade com os seguintes desfechos precoces após o TR: *delirium* (OR 2,05)^84^, função tardia do enxerto (RR 1,78 a 1,80)^8,56^, complicações cirúrgicas (RR 1,88)^8^, prolongamento da internação (RR 1,55)^8^ e readmissão precoce após a alta (RR 1,61)^77,90^.

Entre os desfechos tardios relevantes, pacientes com *status* de fragilidade na admissão do TR apresentam quase o dobro do risco de óbito por todas as causas (RR 1,97), risco que persiste elevado independentemente do tipo de instrumento diagnóstico ou com períodos menores ou maiores de 5 anos de seguimento^8^. Vale ressaltar que, mesmo em pacientes pré-frágeis, há risco 1,5 vez maior de óbito pós-TR^89^. Por outro lado, um estudo americano de coorte retrospectiva com 19.242 pacientes em diálise, que avaliou fragilidade pelo componente físico do SF-36 (SF-12), também observou sobrevida do paciente reduzida (84 vs. 94%) comparando o quartil de valores mais baixos com o mais alto após o TR. Porém, para todos os quatro quartis de SF-12, o benefício de melhor sobrevida foi observado a partir do 9° mês após o TR quando comparado com a persistência em diálise, sugerindo que o benefício de sobrevida após o TR existe nos vários estágios de fragilidade^93^.

Foram relatados também maior intolerância aos imunossupressores, notadamente ao micofenolato^62^, eventos cardiovasculares mais frequentes^77^ e comprometimento da qualidade de vida^86^ (A Figura 1).

## Intervenções

As intervenções para redução da fragilidade em populações com DRC, pré e pós-TR ainda não foram adequadamente estudadas. As experiências aplicadas à população geral podem, entretanto, sinalizar direções a seguir. Devido à natureza multifatorial, estima-se que ações múltiplas poderão atingir melhores resultados. A mensuração dos resultados pode ser avaliada precocemente através das mudanças nos domínios que fazem parte do diagnóstico (como fraqueza muscular, lentidão e exaustão) e, em longo prazo, evitando o declínio e comprometimento funcionais^9^. Para os pacientes em lista, o hiato até o TR representa uma oportunidade de intervenção preventiva, uma vez que o *status* de fragilidade é dinâmico^55^. Por outro lado, após o TR, as medidas poderão modificar os desfechos indesejáveis associados à fragilidade em médio e longo prazos. As medidas propostas incluem reabilitação física, suplementação nutricional, manejo de comorbidades, suporte psicológico e o TR propriamente dito^9,94,95,96,97^.

Publicações prévias reportaram a segurança e viabilidade de intervenções de pré-reabilitação para pacientes em lista para transplante de órgãos sólidos, os quais tiveram como benefício melhora na função cardiorrespiratória, capacidade de exercício, força muscular e qualidade de vida^98,99^. Não há, entretanto, evidências sobre qual seria o melhor conjunto de medidas a ser instituído^97^, exceto duas recomendações institucionais^97,100^. Em relação à atividade física, os estudos são limitados a apenas três publicações que utilizaram diferentes tipos de exercícios (ioga, treinamento de resistência, força e flexibilidade), num período de treinamento de 8 semanas, em amostras pequenas. Entretanto, houve melhora nos componentes de fragilidade, componentes mentais de qualidade de vida e tempo de internação pós-TR^101,102,103^.

O suporte nutricional, para atingir o melhor estado nutricional com recomendações dietéticas por profissional especializado, faz parte do tratamento dos pacientes com DRC, independentemente da inscrição em lista. Entretanto, em pacientes frágeis, uma maior atenção deve ser dada à presença de desnutrição. Intervenções com aumento do aporte calórico e acompanhamento mais amiúde foram utilizadas em transplantes de outros órgãos^97^. Sob o mesmo racional, a otimização dos níveis de hemoglobina seria fundamentada pelo fato de estar associada a inflamação e fragilidade em pacientes incidentes em TR^104^, assim como o melhor controle clínico das comorbidades, como hipertensão arterial, diabetes mellitus, seriam mais relevantes nessa população^95,96^.

As medidas de pré-reabilitação requerem, portanto, estruturação adicional dos serviços e, mesmo na escassez de evidências, estima-se que aumentem a motivação dos pacientes para o TR e que, portanto, funcionem como uma medida de suporte emocional^95^.

O próprio TR é provavelmente a melhor estratégia para melhora do estado de fragilidade. Mesmo nos pacientes mais frágeis é observado o benefício de redução da mortalidade após o TR^93^. Após o estresse cirúrgico, há aumento do risco do surgimento de fragilidade no primeiro mês. Entretanto, a partir do terceiro mês, observa-se melhora dos domínios que compõem a fragilidade e redução acentuada do número de pacientes frágeis^58,60^. Especificamente, há melhora nos domínios de ganho de peso, da força muscular e atividade física^59^. A melhora da uremia é provavelmente o fator modificador pivotal. A prevalência e o risco de tornar-se frágil, no geral, se mantêm menor no primeiro ano^60^, como reportado em estudo brasileiro de Dos Santos Mantovani et al.^61^ e, em longo prazo, após 2,5 anos^59^. Entretanto, por sua natureza multifatorial, o *status* de fragilidade é dinâmico após o TR e em longo prazo, especialmente após 2,5–3 anos há tendência de declínio nos componentes de força e atividade, merecendo maior atenção^59,60^. Considerando as evidências disponíveis, podemos considerar que o status de fragilidade isoladamente não deva ser considerado uma contraindicação para o TR^4,9,93^. Pelo contrário, a inclusão como parte da avaliação pré-TR possibilitará melhor abordagem em lista e após o TR com o objetivo de reduzir os eventos que aumentam a morbidade, uma vez que a avaliação subjetiva sem instrumentos específicos é inacurada em até 37% dos casos^105^.

Os mesmos princípios das medidas de reabilitação são aplicáveis após o TR. Programas visando ao aumento da atividade física, apesar de não avaliarem especificamente o impacto sobre a fragilidade, mostraram eficiência em melhorar a função e o desempenho físico, componentes do fenótipo de fragilidade, assim como melhora na qualidade de vida e de alguns marcadores de doença cardiovascular. Pela falta de evidências robustas, persiste a discussão sobre qual tipo de exercício, intensidade, frequência e duração teria maior benefício^106^. Recomenda-se que se incluam exercícios aeróbicos, combinados ou não com exercícios de resistência, de intensidade moderada a intensa, 3–5 vezes por semana, por, no mínimo, 8 semanas^100^.

## Mensagens Finais

A síndrome de fragilidade é uma condição frequente e subdiagnosticada em pacientes com DRC estágio 5 candidatos ao TR. O subdiagnóstico é consequência da indefinição da ferramenta ideal para o seu diagnóstico e, notadamente, da dificuldade de operacionalizar tal avaliação de forma rotineira e periódica. A fragilidade, cuja etiologia é multifatorial, tem componentes multidimensionais que vão além do fenótipo físico e implicam uma situação de vulnerabilidade mais ampla, com impactos negativos no acesso à lista e ao TR, bem como nos desfechos precoces e tardios após o TR. A necessidade de se estabelecer uma rotina de diagnóstico, manejo e seguimento da fragilidade antes e após o TR é premente, com vistas a melhorar os desfechos desses pacientes^4^.
